# Attitudes of Different Age Groups Toward People With Intellectual Disability During the COVID-19 Pandemic

**DOI:** 10.3389/fpsyt.2021.591707

**Published:** 2021-07-12

**Authors:** Ewa Domagała-Zyśk

**Affiliations:** Department of Pedagogy, The John Paul II Catholic University of Lublin, Lublin, Poland

**Keywords:** intellectual disability Covid-19 pandemic, attitudes towards persons with disability, adults, the elderly, students

## Abstract

**Background:** The COVID-19 pandemic has been particularly risky for people with disabilities and severe medical conditions, not only because the virus may be a direct threat to their physical health but also because of social exclusion and negating their needs and rights.

**Objective:** This study aimed to assess the attitudes of people from different age groups towards people with intellectual disability (ID).

**Methods:** The study included 223 people (19–85 years old) and was conducted in May 2020–January 2021. Data was collected using the *Multidimensional Attitudes Scale Towards Persons With Disabilities* and a self-designed *Questionnaire regarding the attitudes towards people with ID during the pandemic*. Data analysis was performed using SPSS 24 (ANOVA).

**Results:** The results of the study showed that the general attitudes were only slightly supportive and differed among people of different age groups: the youngest and the oldest generation expressed the most positive attitudes while the adults (35–60 y.o.) expressed the most negative ones.

**Conclusions:** As the pandemic is spreading rapidly with no definitive solution, awareness to create more positive attitudes towards people with ID and recognizing their needs is essential.

## Introduction

The COVID-19 pandemic has been especially difficult for the most vulnerable members of society – people with disabilities, the elderly, and those with severe medical conditions. During difficult times like these with limited resources, the true attitudes towards various phenomena and social groups become more visible and prominent. Nowadays, it is not easy to be selfless and sacrifice your time and money for others, if necessary. Patients in many nursing homes have been abandoned, excessive shopping include only the needs of the nearest family members, neglecting the needs of others, and people riot not to express solidarity but to fight for a certain group only. People with disabilities and their caretakes worry that they may not receive good quality healthcare because of their disability (1). This fear has been intensified by global discussions on the need to ration life-saving medical equipment and services, which might be based on wrongly assessed aspects, such as someone's quality of life. As Boyle et al. (1) wrote: “When quality of life, quality-adjusted life years, or disability-adjusted life years are considered in medical rationing decisions, people with disability are unfairly disadvantaged, not because they have lower quality of life, but because they are wrongly assumed to have lower quality of life.”

This study aimed to explore the attitudes of 223 adults towards people with intellectual disabilities in one of the regions of Poland, Central Europe. This study was conducted in first months of Covid-19 pandemic (May 2020–January 2021). Two measures were used: *Multidimensional Attitudes Scale Towards People With Disabilities (MAS-POL)* by Findler et al. (2) in the Polish adaptation of (3) and a self-designed questionnaire on COVID-19 thoughts, emotions, and behavior towards people with intellectual disability (ID) in the pandemic. The results showed differences in people's attitudes, with respondents' age being a critical factor.

## Intellectual Disability and the COVID-19 Pandemic

Intellectual disability (ID) is a lifelong condition that manifests before 18 years of age and involves functional limitations in the areas of learning, language, and behavior (4). Down Syndrome is probably the most common disability associated with ID (5). People with ID have a higher prevalence of comorbidity: hypertension, cardiovascular and lung diseases, obesity, and diabetes (6), which are reported to be risk factors for severe COVID outcomes (7–9).

Adults with ID may live independently, with their family members, or in care home/nursing facilities. In the latter case, everyday life during the pandemic time might seem challenging due to the need of practicing strict hygiene regime and social distancing, which might be difficult to understand and follow by some people with ID. Furthermore, those living in congregated facilities are prone to other risk factors associated with sharing essential facilities (e.g., bathroom or kitchen) with other people, high level of assistance from staff, and multiple shift staffing patterns (10, 11). Some patients might find it difficult to communicate the symptoms of illness, which may delay the diagnosis and allow the virus to spread further (1).

To date, there are only a few reports analyzing case or mortality rates of people with ID or intellectual and developmental disabilities (IDD). The greatest risk of severe COVID-19 outcomes was assessed for people with IDD living in residential group homes. Landes et al. reported that while the case rates for New York general population were 1,919 per 100,000 people, the rates for New York residential homes inhabitants were as high as 7,841 per 100,000 (11). Similar differences could be observed for case fatality (7.9–15%) and mortality rate (151 per 100,000–1,175 per 100,000). In Turk et al.'s report based on TriNetX analysis, it was discovered that COVID-19 was diagnosed more often in younger people with IDD compared to that of in general population (12). The fatality rate for people below 17 years of age with IDD was 1.6 and 0.01% for people without IDD; 4.5% for ages 18–74 years with IDD and 2.7% without IDD. People with ID face additional problems when their routines and support systems are disrupted, which may result in behavior changes (13) or possible loss of function.

Since there is a possibility that COVID-19 pandemic will stay for a long term, not only are the issues of improving health protection efforts and measures vital but long-term goals should also be formulated. One of them is to ensure positive and supportive social attitudes toward people with ID who should not be perceived as a burden to society during the pandemic, but as subjects who should receive good quality medical care and necessary social support and understanding, in accordance with the principles of equality and equity (1). The goal should also be to avoid ubiquitous ableism (14)—prejudice and discrimination against disabled individuals in favor of non-disabled persons—that may affect crisis decisions about medical rationing, other resources allocations and willingness to help and support these in need. As the research shows, pre-pandemic attitudes towards persons with ID were usually not fully positive, but rather negative (15, 16) or neutral (17, 18). They also differ according to participants' age: the attitudes of adults and older persons were usually more negative than these of younger persons (16). In Central European countries it may be due to social and historical issues: educational integration was introduced here after the 1989 transformation, e.g., in Poland in Educationa Act from 1991. Earlier - as in other communist countries - persons with ID were educated in only in segregation institutions and usually also lived there (as they were residential school located in remote areas). This means that older generations usually have not experienced persons with ID neither as schoolmates or neighbors and there were many negative stereotypes about them (19).

Crisis situation like pandemic, connected with personal fears, may strengthen certain attitudes. Ultimately, as COVID-10 pandemic and its consequences enhance the increased risk for abuse among people with disabilities (20), the efforts should be undertaken to assess the risks—among them the attitudes towards persons with ID—and advocate for disability justice and support for person with ID in the pandemic and post-pandemic reality.

## Method

### Aim

This study aimed to assess the attitudes of people of different age groups towards people with ID during the pandemic. Though attitudes towards people with disabilities have been regularly assessed, there are no research analyzing the nature of these attitudes during a crisis.

### Participants and Procedure

The study included respondents aged 19–85 years who volunteered to participate in it. The participants were recruited via a convenience sample: due to classes being conducted online, the university students were staying at their homes and studied online. They were contacted and invited by the researcher to take part in the study and were encouraged to approach their family members, friends, and neighbors to make them participate as well. It should be noted that though the study sample is not large, it includes middle-aged and older persons, not only young ones. Digital skills of older generation in Central Europe are usually poor and it makes it pretty difficult to complete a study sample of that age in online studies - during the pandemic it was the only possible way to collect data. It was assessed that the refusal rate was not more than 10%.

All participants were informed about the details of the study. Study participation was anonymous and voluntary, and participants could withdraw from the study without any consequences. For data-protection reasons, the online survey was open to people aged 18 or over. Only the researchers had access to the research data. The procedures of this study complied with the provisions of the Declaration of Helsinki regarding research on human participants. The research project was approved by the Ethical Committee of the Institute of Pedagogy of the John Paul II Catholic University of Lublin, Poland.

The study was conducted in May 2020–January 2021. The study sample consisted of 223 individuals who were divided into three age groups: young adults aged 19–34 years (*N* = 102), adults aged 35–60 years (*N* = 57), and elderly people aged 61–85 years (*N* = 64). Of these, 81 people were students (36%), 104 worked in the labor market (47%), 8 (4%) were unemployed and 30 (13%) were pensioners. Furthermore, 128 people (57%) lived in towns and 95 (43%) lived in the countryside.

### Instruments

Data was collected using the Polish versions of the *Multidimensional Attitudes Scale Toward People With Disabilities (MAS-POL)* by Findler et al. in the Polish adaptation of (3). The instrument is an indirect measure and uses social scenario vignette to have respondents project their own emotions, thoughts, and behaviors regarding the given situation. The vignette described interaction between three people, male and female, and a person with ID, namely, Down's Syndrome. The respondents were asked to read the vignette and then relate to each item, indicating the degree to which they believed the item reflected how the person in the story would feel, think, or act in the situation. Responses were marked on a 5-point Likert-type scale ranging from 1 (*not at all*) to 5 (*very much*). Polish version of the MAS used in this research was translated and adapted by Byra and Domagała-Zysk (3). Scores were calculated for the participants on each of the factors by averaging participants' scores on the items of each subscale; higher scores represented more negative attitudes towards people with disabilities (items phrased in the opposite direction were reverse coded for the purpose of this analysis).

The second tool used was *Attitudes towards persons with intellectual disability during the Covid-19 pandemic*. It consisted of six social stories describing life-like situations of the pandemic, such as difficulties in social distancing, possible job reduction, shortage of medical equipment for COVID-19 patients, queues for rehabilitation services, and stronger need for social benefits when the sources are not sufficient. Each story was accompanied by three reactions, and they were rated by the respondents on a 7-point Likert-like scale. The responses expressed positive or negative behavior, such as “*People with ID should not be allowed in public spaces as they do not respect the rules of social distancing*” or “*People with ID should not be given additional social benefits as this money could be spent on other needs.*” One third of the responses were neutral. The process of designing the tool was two-fold; in the first stage, “pandemic stories” were collected from a focus group via online meeting consisting of university students in the final year of Special education program. A total of 48 stories were collected and subsequently analyzed by three experienced researchers in disability. The final set of six stories with three reactions each was designed. The set was then assessed by three other researchers; among them were people with motor or sensor disabilities. The content and language of the stories were adjusted to describe best the pandemic possible behavior of general audience. After collecting the data, the responses to the stories were counted (some of them were reversely coded), and a final result was obtained from every participant that indicated *positive (36*–*48 points), neutral (24*–*35) or negative (23 or less)* attitude towards people with ID during the pandemic.

The two abovementioned scales were accompanied by a demographic chart that collected participants' age, sex, education level, employment status, and past experiences with people with ID.

Data analysis was performed using SPSS 24 (ANOVA).

## Results

The results showed significant differences between the age groups concerning attitudes towards people with ID: attitudes of adults (35–60 years) were significantly more positive in the emotional aspect than those of the young adults (35–60 years; *p* < 0.05) and more positive in the cognitive aspects than these of the elderly participants (*p* < 0.05, Table 1). When the oldest respondents were asked about their experience with people with ID, they mostly reported feelings of shame, distress, or guilt.

**Table 1:** Descriptive statistics for the group (*n* = 223) with age as a grouping variable.

**Dimension of attitudes**	**Group I (19–34 y.o.)** ***N*** **=** **102**		**Group II (35–60 y.o.)** ***N*** **=** **57**		**Group III (61–85 y.o.)** ***N*** **=** **64**		**ANOVA**		**Significant differences between groups**
	**M**	**SD**	**M**	**SD**	**M**	**SD**	***F***	***P***	
Emotions	43.46	9.78	39.23	9.49	42.34	11.36	3.191	0.043	I-II (*p* = 0.034)
Thoughts	27.88	9.67	26.33	9.52	30.58	8.77	3.238	0.041	II-III (*p* = 0.036)
Behavior	23.95	6.31	23.46	6.85	22.66	5.69	0.834	0.436	

Further analysis was performed to check the consistency of the attitudes. Consistency was found between emotions and behavior in all three groups (*p* < 0.01) and between emotions and thoughts in the group of adults (35–60 y.o., Table 2). It was thus observed that adults present the most consistent pattern of attitudes towards persons with ID, especially in the dimensions of emotions, thoughts and behavior.

**Table 2:** Consistency of attitudes as measures by MAS-POL for participants of different ages (*n* = 223).

**Correlations**	**Group I (19–34 y.o.)** ***N*** **=** **102**		**Group II (35–60 y.o.)** ***N*** **=** **57**		**Group III (61–85 y.o.)** ***N*** **=** **64**	
	**rho**	***p***	**rho**	***p***	**rho**	***p***
Emotions-thoughts	−0.020	0.838	−0.283^*^	0.033	−0.205	0.104
Emotions-behavior	0.501^**^	0.000	0.557^**^	0.000	0.442^**^	0.000
Thoughts-behavior	0.081	0.421	−0.066	0.624	−0.164	0.195

* *p < 0.05*,

** *p < 0.01*,

and ^***^p < 0.001.

A significant trend was observed when the attitude towards people with ID during the pandemic was measured. The results showed that the attitudes of persons of all generations towards people with ID were neutral – *neither negative nor positive*. Interestingly, there were significant differences between the responses of the oldest and the youngest participant and adults, whose attitude was the most negative (*p* < 0.05, cf also Table 3).

**Table 3:** Attitudes towards people with ID during pandemic (*n* = 223).

**Attitudes towards people with ID during the pandemic specific situations**	**Group I (19–34 y.o.)** ***N*** **=** **102**		**Group II (35–60 y.o.)** ***N*** **=** **57**		**Group III (61–85 y.o.)** ***N*** **=** **64**		**ANOVA**		**Significant differences between groups**
	**M**	**SD**	**M**	**SD**	**M**	**SD**	***F***	***P***	
COVID-19 questionnaire	31.4	6.16	28.82	5.67	33.16	6.32	7.695	<0.001	I-II (*p* = 0.030) II-III (*p* < 0.001)

## Discussion

Social justice based on humanistic values in democratic societies imposes an obligation to take care of these vulnerable, especially in difficult crisis situation. As researchers notice, person's attitudes convey their present and future decisions that might offer support – or deny it. In the pre-pandemic research it was often concluded that non-disabled people hold negative attitudes towards people with disabilities (3, 18, 21, 22), especially towards persons with intellectual challenges (15, 16). This is also true for professionals, e.g. health care providers (23, 24), who tended to underestimate the quality of life of persons with disabilities and be pessimist about their future.

As the pandemic is spreading worldwide, the increasing risk to people with ID is evident. Biased attitudes towards this population, lack of professional advocacy and expressions of group solidarity create a direct danger to persons with ID well-being, quality of life and even their right to proper medical care.

Attitudes towards persons with ID in the pandemic reality is a kind of a test of solidarity and humanistic values employed in practice. The present results mirror these risks and show that attitudes towards people with ID during the COVID-19 pandemic are ambivalent: *neither positive nor negative*. This may be interpreted as a lack of overt hostility or exclusive behavior; however, these are not attitudes that represent care, support, or understanding as well. The results are similar to the pre-pandemic results of Papuda-Dolińska (17) or Pace et al. (18) where adults' attitudes towards persons with disabilities were assessed as *moderate* and Byra and Domagała-Zysk (3) who stated that many adults hold negative attitudes towards persons with ID.

In our study the most positive attitudes were expressed by the youngest – and the oldest generation. This indicates that young people, who received integrative and inclusive education at every stage of their schooling (integration in education started in Poland only in 1989) and experienced more prominent presence of people with ID in their social environment, presented more positive attitudes towards people with ID in the crisis situation. These results are consistent with Morin et al. (16): in their studies more positive attitudes were revealed among younger participants. If we compare these results with the pre-pandemic research on attitudes towards persons with disabilities in Poland where the MAS instrument was also used [(3), study conducted in 2019, *N* = 562, respondents' average age = 21] it can be noticed that they share many similarities. In both studies the attitudes were moderately positive, low in direct hostility, shame or distress, but lacking positive emotions of calmness, serenity or relaxation in possible direct contact with persons with disabilities.

The oldest generation appeared to be the most compassionate towards persons with ID in the pandemic time. This may be due to the fact they understand their situation as similar – both the disabled and the elderly were most vulnerable to the negative effect of SARS-Cov-2 both in the medical sense and also in the social dimension.

People with intellectual disabilities have experienced systemic ableism (25) and the pandemic intensifies risks, stress and trauma they experience. As the attitudes towards persons with intellectual disabilities are not fully positive, they call for action and further activities on changing them for the future: it must be clear that disabled lives matter to us, also in time of threat. Though the issue of a limited number of lifesaving equipment and care services is no longer a problem in many countries as the pandemic is under control, there is still a strong need to monitor the situation and advocate for people with ID. As the economic crisis deepens, some of the services might be threatened and the tendency (visible in the research results) to not support one of the most vulnerable groups may have been further broadened. There surely will appear new solutions that may be limited at a certain point, like a new pharmaceutical or vaccine. As Boyle et al. (1) postulates, people with disabilities should be among the first to receive these treatments. Medical services or good rationing decisions in no case should be based on disability status [cf. (14)]. Moreover, we should recognize the needs of people with ID for meaningful social interactions to maintain better mental health and well-being while self-isolating. Non-disabled people with strong positive attitudes towards people with ID seem to be vital during this process.

## Limitations

The present study, although it provides valuable results in a mixed-aged group of respondents who participated in the study in the first months of SARS-Coc-2 pandemic, is not free from limitations. First, the study sample does not allow for generalizations and a bigger research should be planned to get the representative results. Secondly, only the basic demographic data were collected. It might be beneficial to analyze in detail the attitudes of participants who differ in their socioeconomic, educational or professional status. However, for some participants in our country (as the researchers' experience proves) it is uncomfortable to disclose personal information. What is more, the educational level of the country's population has dramatically changed – from 14.4% in 2002 to 45.7% in 2019. This means the educational characteristic should be analyzed within age groups and this requires even bigger sample. Thirdly, the research focuses on a selected elements on the pandemic situation which is dynamic, so both the conditions and attitudes might be fluctuating.

## Data Availability Statement

The raw data supporting the conclusions of this article will be made available by the authors, without undue reservation.

## Ethics Statement

The studies involving human participants were reviewed and approved by Ethical Committee of the Institute of Pedagogy of the John Paul II Catholic University of Lublin, Poland. The patients/participants provided their written informed consent to participate in this study.

## Author Contributions

ED-Z responsible for the research design, statistics, and writing the paper.

## Conflict of Interest

The author declares that the research was conducted in the absence of any commercial or financial relationships that could be construed as a potential conflict of interest.
